# Combining Old and New Tricks: The Study of Genes, Neurons, and Behavior in Crayfish

**DOI:** 10.3389/fphys.2022.947598

**Published:** 2022-07-06

**Authors:** Wolfgang Stein, Margaret L. DeMaegd, Abigail M. Benson, Rajit S. Roy, Andrés G. Vidal-Gadea

**Affiliations:** ^1^ School of Biological Sciences, Illinois State University, Normal, IL, United States; ^2^ Stiftung Alfried Krupp Kolleg Greifswald, Greifswald, Germany; ^3^ Center for Neural Sciences, New York University, New York, NY, United States

**Keywords:** marbled crayfish, CRISPR, tail flip, stomatogastric ganglion, marmorkrebs, giant fiber, RNAi, transgenesis

## Abstract

For over a century the nervous system of decapod crustaceans has been a workhorse for the neurobiology community. Many fundamental discoveries including the identification of electrical and inhibitory synapses, lateral and pre-synaptic inhibition, and the Na^+^/K^+^-pump were made using lobsters, crabs, or crayfish. Key among many advantages of crustaceans for neurobiological research is the unique access to large, accessible, and identifiable neurons, and the many distinct and complex behaviors that can be observed in lab settings. Despite these advantages, recent decades have seen work on crustaceans hindered by the lack of molecular and genetic tools required for unveiling the cellular processes contributing to neurophysiology and behavior. In this perspective paper, we argue that the recently sequenced marbled crayfish, *Procambarus virginalis*, is suited to become a genetic model system for crustacean neuroscience. *P. virginalis* are parthenogenetic and produce genetically identical offspring, suggesting that germline transformation creates transgenic animal strains that are easy to maintain across generations. Like other decapod crustaceans, marbled crayfish possess large neurons in well-studied circuits such as the giant tail flip neurons and central pattern generating neurons in the stomatogastric ganglion. We provide initial data demonstrating that marbled crayfish neurons are accessible through standard physiological and molecular techniques, including single-cell electrophysiology, gene expression measurements, and RNA-interference. We discuss progress in CRISPR-mediated manipulations of the germline to knock-out target genes using the ‘Receptor-mediated ovary transduction of cargo’ (ReMOT) method. Finally, we consider the impact these approaches will have for neurophysiology research in decapod crustaceans and more broadly across invertebrates.

## Introduction

### The History

For more than 70 years, the thoroughly studied nervous system of decapod crustaceans has been a workhorse for a large segment of the neurobiology community. Approximately one hundred laboratories currently work on the nervous system of decapod crustaceans, on a variety of topics that range from cellular and synaptic processes to learning, memory, and behavior. Electrophysiological experiments in the crustacean nervous system have yielded many fundamental discoveries. These include the discovery of the Na^+^/K^+^-pump (Skou, 1957) and electrical and inhibitory synapses (Farca Luna et al., 2010; Jirikowski et al., 2010), and the characterization of fundamental aspects of synaptic function. Early works on endplate potentials at the neuromuscular junction (Katz and Kuffler, 1946; Fatt and Katz, 1953) and on the role of GABA as an inhibitory transmitter (Bowery and Smart, 2006) were instrumental in understanding the basics of chemical synaptic transmission in all animals. The identification of connectivity, modulators, and transmitter systems in several crustacean species (Skiebe, 2001; Nusbaum and Beenhakker, 2002; Skiebe, 2003; Daur et al., 2016; Stein et al., 2016; Stein, 2017) revealed many principles of neuronal functioning, including lateral inhibition and the processing of visual stimuli (Hemmi and Tomsic, 2012; Zieger et al., 2013; van Oosterhout et al., 2014), presynaptic inhibition of sensory receptors (Soedarini et al., 2012), neuromodulator actions (Stein, 2009; Nusbaum and Blitz, 2012), coordination of neural circuits (Mulloney and Smarandache-Wellmann, 2012), network dynamics (Nadim and Bucher, 2014), the generation of rhythmic motor activity (Marder, 2000; Nusbaum and Beenhakker, 2002; Selverston et al., 2009), the control and selection of stereotyped behaviors by modulatory command neurons (Edwards et al., 1999; Nusbaum and Beenhakker, 2002; Marder and Bucher, 2007; Stein, 2009; Harris-Warrick, 2011), and recently the characterization of degenerate circuits (Marder and Rue, 2021).

Many factors contribute to the enduring usefulness of decapod crustaceans for the study of neural function and behavior. A particular advantage is that the principles of neuronal operation can be studied in circuits that are specialized to specific behaviors and often built from only a few neurons. A given ganglion, such as the stomatogastric ganglion, for example, controls multiple behaviors with its ∼30 neurons, including the chewing and subsequent filtering of food. As such, the number of cells committed to a single behavior can be even further reduced. Crustacean neurons also tend to be large, with somata reaching up to 100 μm in diameter. This provides unique access to the cell-intrinsic physiology and facilitates otherwise challenging intra- and extracellular electrophysiological recordings. Many neurons are even uniquely identifiable between individuals and have well-defined homologs in related species. This enables comparative studies of neuronal function and behavior at the single cell level (Schmidt-Rhaesa et al., 2015; Stein et al., 2016). It also allows studies that address evolutionary changes to the function of individual neurons—a feature essentially absent in studies of brains with more neurons. Finally, whole circuits can be isolated from the animal and kept alive for up to several weeks (Luther et al., 2003; Sakurai and Katz, 2009) with minimal saline and superfusion requirements. As a result, network reconstruction and homeostatic mechanisms of repair and re-organization after nerve injury can be analyzed at the cellular and circuit levels. Neurons can also be dissociated and placed in cell culture for physiological and molecular analyses (Turrigiano et al., 1994).

### The Problem

While decapod crustaceans have excelled in studies of neuronal physiology, research has made slow progress in the adoption of genetic and molecular tools. Previous studies were mostly restricted to gene expression measurements and system-wide suppression of genes through RNA interference (RNAi) (Sagi et al., 2013; Northcutt et al., 2016), with few exceptions (Dearborn et al., 1998; Yazawa et al., 2005; Posiri et al., 2013). That resulted in the conundrum that the activities and circuits that underlie many fundamental behaviors are well-characterized, but genetic and molecular insights into neural functioning are often missing. For example, a paradigm-shifting finding from the crustacean stomatogastric ganglion is that within a neuronal circuit, multiple combinations of intrinsic and synaptic conductances can lead to the same functional output. Models of the crab stomatogastric nervous system have long predicted that this is the case (Prinz et al., 2003). Experimental evidence from the same system supports the model predictions, showing substantial variability in ion channel expression levels between individuals of the same species and between neurons of the same type (Schulz et al., 2006; Golowasch, 2019). This variability is degenerate, as its effects are not observable unless the system is challenged by extreme or pathological conditions. It is under these conditions where circuits and their behaviors become unpredictable, and differences between individuals are revealed. While the stomatogastric nervous system is one of the best studied crustacean nervous tissues (Stein, 2017), establishing cause-and-effect relationships between genes and neuron physiology has proven challenging. For instance, despite numerous studies on this topic, we are no closer today to having an answer to the origins of circuit parameter variability, nor do we understand how this variability is controlled. It is for this reason that others have called for establishing a new model system to study the molecular and genetic underpinnings of neuronal function in decapod crustaceans (Schulz and Lane, 2017).

In genetic model systems, neurons and circuits can be identified with genetic markers, recorded with optical imaging, and manipulated with light-induced ion channels and pumps, for instance. No doubt that pairing these techniques with the outstanding access to the behavioral performance of these animals has opened many new avenues to study neuronal function, behavior, and neuropathologies. Yet, solely by themselves, these techniques are unable to provide access to many of the neuronal and circuit dynamics that are crucial for neuronal functioning. Even the most sophisticated molecular techniques are unable to capture changes in membrane conductance or input resistance, for instance. Routine electrophysiology from intact circuits is challenging in many genetic model systems, and this absence of physiological tractability remains an obstacle for understanding how neuronal and circuit dynamics interact to generate behavior.

### Perspective: Combining Old and New Tricks Through The Study of Genes, Neurons, and Behavior in Crayfish

**FIGURE 1:**
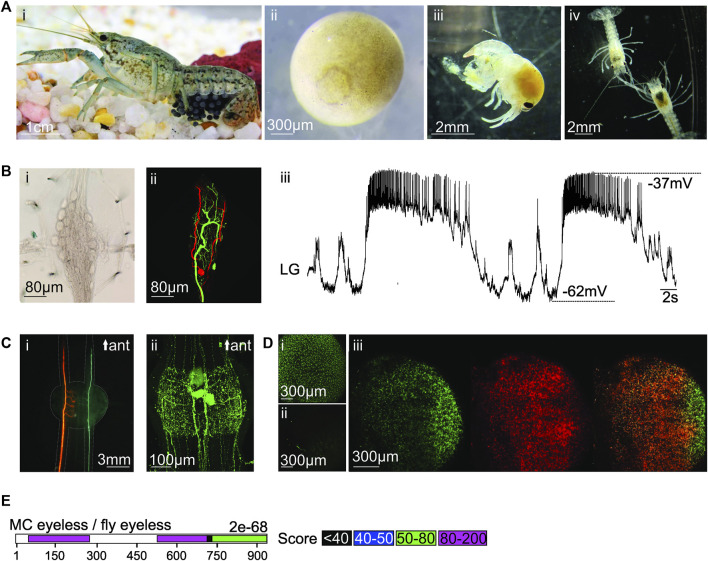
**(A)** (i) Photo of pregnant marbled crayfish. Credits: Carola Städele. (ii) Magnification of stage 2 oocyte (10–15% development) with circular blastophore. (iii) Freshly hatched juvenile (stage 1). (iv) Two translucent stage 2 juveniles. Digestive tract and hepatopancreas are clearly visible. **(B)** (i) Brightfield image of adult stomatogastric ganglion with neuronal somata of the pyloric and gastric mill central pattern generators. (ii) Confocal image of two stained stomatogastric neurons. Somata, axons and neuropil are visible. (iii) Intracellular recording of lateral gastric (LG) neuron in the stomatogastric ganglion. Signal to noise ratio and waveform of the membrane potential oscillations are similar to other crustaceans (Stein et al., 2022). **(C)** (i) Staining of the two bilaterally symmetric lateral giant neurons in the marbled crayfish ventral nerve cord. (ii) Immunohistochemical staining against Serotonin in a marbled crayfish ventral nerve cord ganglion. **(D)** (i) Oocyte containing GFP after ReMOT treatment. (ii) A control oocyte shows no GFP staining. (iii) Overlay of GFP (left) and anti-GFP immunohistochemistry (middle) in oocyte. Right: Yellow delineates overlap in GFP and anti-GFP fluorescence signals. **(E)** Sequence alignment between putative MC eyeless and fly eyeless transcripts. Colored bars: significant alignment with scores between the two sequences. Overall e-value is provided.

The marbled crayfish, *Procambarus virginalis* (Figure 1Ai)*,* has the potential to innovate how we study neuronal function and behavior, because it allows integrating genetic and molecular insights with circuit and cellular physiology. It has recently joined the list of sequenced animals with a high coverage genome and transcriptome (Gutekunst et al., 2018), complementing and expanding the list of sequenced decapods (Kenny et al., 2014; Song et al., 2016; Yuan et al., 2018). Unlike its previously studied relatives that are typically wild-caught, *P. virginalis* fulfills standard requirements for laboratory culture. It exhibits robustness against handling stress and is inexpensive to house and upkeep. Marbled crayfish start to reproduce 90–140 days after hatching, with breeding periods of 21–37 days, and interclutch periods of 50–85 days (Seitz et al., 2005). Clutch sizes can reach several hundred eggs (Vogt, 2010) with a hatching success rate of up to 80% (Seitz et al., 2005; Vogt, 2008).

Importantly, for genetic studies, all individuals are female triploids (Vogt et al., 2015) that produce genetically uniform offspring *via* apomictic parthenogenesis—oocytes develop without fertilization or meiosis (Seitz et al., 2005; Martin et al., 2007; Vogt, 2010; 2011). In case of new strains or mutants, this preserves the introduced manipulation without the need for outcrossing. Maintaining even only a few adult animals can ensure a steady supply of eggs for genetic manipulations as an individual can complete up to 7 reproductive cycles during its lifetime (Vogt et al., 2004). Eggs are large (∼1.6 mm diameter, Figure 1Aii), develop externally, and can be raised without maternal care (Henryon and Purvis, 2000; Leonard et al., 2001; Seitz et al., 2005). The fact that all developmental stages are easily accessible for experimental manipulation offers many advantages over the mammalian intrauterine development or metamorphosis in other arthropods, such as flies. Importantly, eggs, embryos, and juveniles (Figures 1Aiii,iv) are translucent and have a high tolerance to physical manipulation (Vogt, 2007), so they can easily and rapidly be assayed for naturally occurring or experimentally-induced phenotypes. This includes defects in patterning and limb formation that can be assessed because of the slow short-germ development, i.e., the sequential addition of body segments during morphogenesis.

The marbled crayfish nervous system shares many similarities with other decapod crustaceans. For example, the architecture of the embryonic CNS of marbled crayfish and the Australian crayfish (*Cherax destructor*) are strikingly similar (Carr and Boudreau, 1996), including their aminergic and peptidergic transmitter systems (Polanska et al., 2007; Rieger and Harzsch, 2008). Our data show that their stomatogastric ganglion has large identifiable neurons that are similar in morphology (Figures 1Bi, ii) and activity patterns to lobsters, crabs and other crayfish (Figure 1Biii). Similarly, the medial and lateral giant neurons in the ventral nerve cord (Edwards et al., 1999) are easily identifiable (Figure 1Ci), and aminergic neurons can be stained (Figure 1Cii). Even as juveniles, animals and neurons are large enough for electrophysiological investigations. Juveniles are also translucent, which permits the visualization of labeled cells and optogenetic control of neural function. Importantly, crayfish, including marbled crayfish, have a rich repertoire of behaviors that make them well-suited for neuroethology and for studying the underlying causes of behavioral symptoms of diseases. The brilliant work of Huxley at the end of the 19th century already described many crayfish behaviors, their natural history and basic physiology, and provided guidance and concepts for future researchers (Huxley, 1880). Today, ethograms of many crayfish escape (Edwards et al., 1999; Herberholz and Marquart, 2012) and social behaviors (Heckenlively, 1970; Copp, 1986; Figler et al., 1995; Yeh et al., 1997; Issa et al., 1999; Vorburger and Ribi, 1999; Herberholz et al., 2001; Baird et al., 2006) have been developed and the underlying circuits are known to varying degrees. This has already established them as model organisms for social dominance (Edwards et al., 2003; Abe and Nagayama, 2021), anxiety (Fossat et al., 2014), intoxication (Swierzbinski et al., 2017; Venuti et al., 2021), and decision making (Herberholz and Marquart, 2012). Crayfish can learn to perform tasks and have been used to study learning and memory (Krasne and Bryan, 1973; Kawai et al., 2004). Finally, crayfish have been used for studies ranging from kinematics and locomotion to the neuronal control and coordination of behavior (Farca Luna et al., 2009), suggesting that they could be well-suited for studying the wide variety of genetic disorders that affect motor control.

Marbled crayfish may be a suitable middle-ground between small invertebrates where behaviors are often measured as a population response, and larger vertebrates where single animals are studied. However, there is still a major need to establish reliable methods to drive or suppress gene expression, and to incorporate molecular tools that allow target-specific manipulation of identified neurons and circuits. We suggest that the first steps could include the expression or knock-out of genes involved in brain functioning or development that could be monitored by the resulting morphological and behavioral changes.

### Transgenesis to Manipulate Gene Expression in Marbled Crayfish Neurons

Their apomictic parthenogenesis makes marbled crayfish uniquely suited for the adoption of transgenesis. However, there are no established protocols to introduce genetic constructs into marbled crayfish cells or oocytes. Indeed, we found that iontophoretic and pressure injections of Green Fluorescent Protein (GFP) plasmids into freshly laid eggs were unsuccessful. Similarly, attempts to electroporate the GFP plasmid failed. Eggs either died within a day after electroporation or did not incorporate the plasmid.

To address these issues, we are currently pursuing the “Receptor-mediated ovary transduction of cargo” (ReMOT) method, which exploits the vitellogenesis pathway in oviparous species to transport synthetic molecular cargo into developing oocytes (Chaverra-Rodriguez et al., 2018). This approach was developed to provide a high-throughput and simple way to transport genetic constructs into oocytes, with the potential to affect an entire clutch of offspring. Pregnant animals are injected with the ReMOT protein and its cargo, which is then transported into the oocytes. We have already shown that in an *in vitro* setting, where eggs were raised without the mother, GFP was successfully transported into the oocytes. Figures 1Di,ii show a comparison of one egg treated with ReMOT (i) and a control egg (ii). GFP fluorescence was only present in the ReMOT treated egg. To further verify the presence of GFP inside the oocyte, we carried out anti-GFP immunohistochemistry (Figure 1Diii). There was good overlap between GFP (green) and anti-GFP signals (red) as indicated by the overlay (yellow). We also found that marbled crayfish survive the injection and lay healthy eggs. Similar results have been reported for the transport of mCherry into oocytes (Lyko, personal communication).

We now aim to achieve stable CRISPR/Cas-9 gene edits that result in easily observable, morphological deformities that can be detected early in development, such as abnormal eye or limb development. As a first step, we have identified the putative homolog of the drosophila homeobox gene *eyeless* (Figure 1E), which controls early eye development and has already been demonstrated to alter eye phenotypes in crustaceans (Nakanishi et al., 2015).

## Discussion

Work on decapod crustaceans has been pivotal for our understanding of basic processes of neural function and has shed light on processes in virtually all areas of cell and systems biology. We argue that the marbled crayfish is ideally suited to become a new genetic model for neurophysiology and behavior. Above all, the basic neurosciences field will benefit from new tools to study the genetic basis of neural function. Combining the extensive knowledge base available for decapod crustaceans with these molecular techniques will reveal how physiological processes interact with the molecular cell machinery. The comparably large crayfish neurons allow the design of hierarchical, step-by-step experimental approaches to study neuronal processes across levels of organization, linking the molecular machinery of individual neurons with the dynamic organization of complex nervous systems. There are very few systems that allow this level of analysis.

What are initial questions and projects that would benefit from having better genetic and molecular tools available? Marbled crayfish neurobiology is currently a wide-open field, and the number of publications is still very modest. Early work focused on the development of the nervous system and some of its neurotransmitters (Vilpoux et al., 2006; Polanska et al., 2007; Fabritius-Vilpoux et al., 2008; Rieger and Harzsch, 2008). More recently, studies have investigated adult neurogenesis (Brenneis et al., 2021), operant conditioning (Okada et al., 2021), phototaxis (Shiratori et al., 2017), and psychostimulant actions on marbled crayfish behavior (Jackson and van Staaden, 2019). However, most of these studies used traditional non-genetic methods.

### Innovating the Classical

Arguably one of the best-characterized neuronal circuits is the one controlling the crayfish lateral giant (LGi) tail-flip escape response (Edwards et al., 1999; Sillar et al., 2016). In this behavior, the LGi neuron (Figure 1Ci) initiates a powerful and highly stereotyped abdominal contraction that moves the crayfish upwards and away from the source of threatening stimuli. The underlying neuronal circuit comprises several rectifying electrical synapses, for example between the primary mechanosensory afferents in the tail fan and the LGi neuron and between the LGi neuron and the giant motor neurons. Enabling the rapid and feed-forward flow of electric current from sensory neurons to motor neurons, these rectifying electrical synapses lead to a rapid contraction of the anterior abdominal muscles and the escape tail flip. In fact, it was in the LGi escape circuit that the first polarized transmission between electrically coupled cells was discovered (Furshpan and Potter, 1959).

How do electrical synapses achieve their rectifying properties? Do giant fibers express distinct gap junction isoforms, and, if so, which ones? How does the escape behavior change if synapses are not rectifying or if rectification is weakened or strengthened? Answering these questions would certainly improve our understanding of crayfish escape responses but may also inform hypotheses across systems by providing insight into gap-junction mediated neuronal synchrony, for example. Studying the function and modulations of gap junction proteins requires access to genes and transcripts, and ways to manipulate gene expression in addition to the established electrophysiological techniques.

**FIGURE 2:**
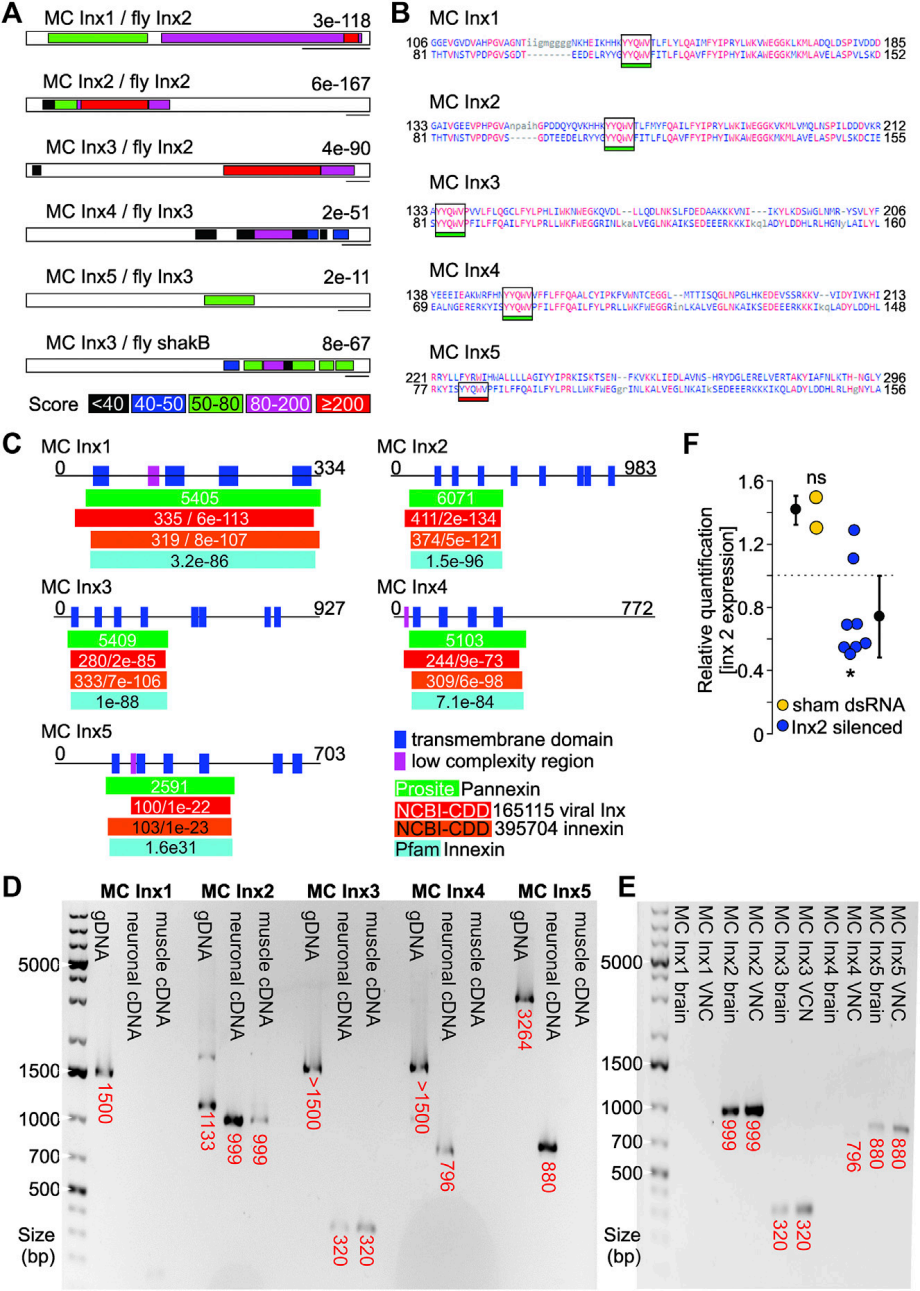
**(A)** Blast comparison of marbled crayfish (MC) putative innexin transcripts and identified innexins from *Drosophila melanogaster*. E-values are given. Colors indicate score. Scale bars: 200 bp. **(B)** Amino acid projection of putative MC innexins (top) and innexin amino acid sequences of a domain model from other species (bottom). MC innexins 1-4 are predicted to contain the innexin hallmark YYQWV amino acid sequences. **(C)** Motif and domain prediction of putative MC innexin amino acid sequences. All five innexins contain transmembrane domains and include known innexin motifs in their amino acid sequence (colored bars). **(D)** and **(E)** Gel electrophoresis of innexin sequences amplified using intron spanning PCR primers. **(D)** Innexins 2-5 had abundant expression in neuronal tissue. Innexins 2 and 3 were additionally expressed in muscle tissue. mRNA was extracted from neuronal tissue (brain, abdominal, and thoracic ganglia) and from abdominal muscles, respectively. **(E)** Innexins 2, 3, and 5 showed abundant expression in the brain. Innexins 2, 3, 4, and 5 were expressed in the ventral nerve cord. **(F)** RNAi-induced reduction of MC innexin 2, 2 days after treatment with dsRNA. Scrambled dsRNA was used for sham injections. *p < 0.05, ns = not significant, one sample t-test against housekeeping gene (EIF-α).

Using sequence homology with the well-characterized Drosophila innexins, we have identified five putative gap junction gene homologs (Figure 2A). Four of them encode the YYQWV amino acid sequence (Figure 2B), a known conserved domain of invertebrate gap junction proteins (called innexins) that is consistently found in their transmembrane domain (Yen and Saier, 2007). Innexins are known to contain 4 transmembrane domains (Phelan et al., 1998), and we found at least 4 transmembrane domains in each marbled crayfish putative innexin sequence (Figure 2C). Additionally, mRNA extractions indicate the putative innexins 2–5 are expressed in neuronal tissue (Figure 2D). Innexins 2, 3 and 5 are expressed in the brain, while innexins 2, 3, 4, and 5 are expressed in the ventral nerve cord (Figure 2E). We have also shown that RNAi reduces innexin 2 expression 2 days after treatment (Figure 2F). Which innexins contribute to the tail flip circuits can now be identified by monitoring changes to the tail flip behavior with high-speed video recordings.

Similar genetic screening approaches can be used to tease out the role of neurotransmitter and modulator receptors. Serotonin, Dopamine, and GABA have well-described influences on the tail flip circuit, and implications in social behaviors like aggression and dominance hierarchy (Edwards et al., 2003). Some Serotonin receptors have been cloned in crayfish (Sosa et al., 2004), but their role in tail flip, dominance and aggression circuits has not specifically been examined. What are the functional roles of distinct receptor subtypes and especially within the context of escape and social interactions? Are particular subtypes expressed together, or in particular contexts? Integrating genetic and electrophysiological techniques in marbled crayfish would allow us to approach these questions.

### Expanding the New

The concept of degenerate circuits, i.e., the hidden variability of neuronal and synaptic parameters between individuals, is the topic of many recent studies in the stomatogastric nervous system (Marder and Rue, 2021). How this variability comes about and how it is controlled is unknown. Degenerate variability could be caused by three, non-mutually exclusive possibilities: 1) stochastic events in regulatory genes that control ion channel expression, 2) life-history-dependent homeostatic plasticity, or 3) regulatory genes differ between individuals, as well as between cell types. Marbled crayfish are well-suited to address the question of the origin of this variability. Their stomatogastric neurons are homologous to previously studied species and offer the same cellular access to their intrinsic and synaptic properties (e.g., Figure 1B). In contrast to species with diverse genetic background and unknown life histories, such as wild-caught crabs and lobsters that were used for previous studies, life history can be fully controlled in marbled crayfish, and genetic diversity is mostly absent. There are already established protocols for the identification and annotation of neuronal genes, as demonstrated by the recent characterization of two putative GABA-A receptor subunits in marbled crayfish (Stein et al., 2020). Gene expression measurements in crustacean tissues and even single cells have long been established (Schulz et al., 2007). While the latter were not developed for marbled crayfish, we have recently demonstrated that RT-PCR and qPCR of neuronal genes are feasible using primers derived from the marbled crayfish genome and transcriptome (e.g., Figures 2D,E).

### Into the Future

Marbled crayfish continue to produce new neurons throughout their life [reviews: (Vogt, 2008; Sandeman et al., 2011; Vogt, 2018; Wittfoth and Harzsch, 2018)]. There are two major sites of new neuron production in the crayfish nervous system. The first is in a specialized proliferation center called the neurogenic niche in the crayfish brain that contains neuronal precursor cells (Sullivan et al., 2007; Sintoni et al., 2012). Resembling the situation in vertebrates, precursor cells migrate to their destination sites where they give rise to new neurons. The second site where neurons are added is the olfactory system. The outer branches of the first antennae bear the olfactory aesthetascs, each of which contains about 100 bipolar receptor neurons (Sandeman and Sandeman, 2003). Juvenile marbled crayfish have only about 10 aesthetascs, but in each moulting cycle additional aesthetascs are added, so that adults end up with several hundreds of them (Vogt et al., 2004). The axons of the new sensory neurons must find their way to the brain, where they must be integrated into the already existing neuronal network of the olfactory lobe. Most recently, first insight into the genetic regulatory network underlying neurogenesis has been provided (Brenneis et al., 2021), but generally speaking, very little is known about the molecular, genetic, and physiological profiles of these processes. Further combining these approaches in marbled crayfish will open new avenues to investigate neurogenesis, axonal path-finding, and regeneration of neurons.

### Roadblocks

There are still many challenges to achieve the goal of establishing the marbled crayfish as a new genetic model system for neurophysiology. For example, marbled crayfish are triploid. This makes the identification and validation of genes involved in neuronal function slow and cumbersome, as more controls are needed to ensure the success of potential gene manipulations. With methods to manipulate gene expression still in their infancy and established protocols from other animals often failing or being difficult to adapt, it will require the collaborative effort of many laboratories to move forward. However, with the ever more rapid development and availability of new genetic techniques, such as CRISPR and viruses-mediated genome editing, these challenges are likely to be overcome quickly.

## Data Availability

Nucleotide sequence data reported are available in the Third Party Annotation Section of the DDBJ/ENA/GenBank databases under the accession numbers TPA: BK061407-BK061411. Additionally, the data for this study have been deposited in the European Nucleotide Archive (ENA) at EMBL-EBI under accession number PRJEB53983 (https://www.ebi.ac.uk/ena/browser/view/PRJEB53983). Publicly available datasets were also analyzed in this study. These data can be found here: https://www.ncbi.nlm.nih.gov/assembly/GCA_020271785.1/
